# Floods and diarrheal morbidity: Evidence on the relationship, effect modifiers, and attributable risk from Sichuan Province, China

**DOI:** 10.7189/jogh.12.11007

**Published:** 2022-07-25

**Authors:** Tianjiao Lan, Yifan Hu, Liangliang Cheng, Lingwei Chen, Xujing Guan, Yili Yang, Yuming Guo, Jay Pan

**Affiliations:** 1 HEOA Group, West China School of Public Health and West China Fourth Hospital, Sichuan University, Chengdu, China; 2 Institute for Healthy Cities and West China Research Center for Rural Health Development, Sichuan University, Chengdu, China; 3 School of Public Health, Sun Yat-sen University, Guangzhou, China; 4 Sichuan Center for Disease Control and Prevention, Chengdu, China; 5 Climate, Air Quality Research Unit, School of Public Health and Preventive Medicine, Monash University, Melbourne, Australia

## Abstract

**Background:**

Although studies have provided the estimates of floods-diarrhoea associations, little is known about the lag effect, effect modification, and attributable risk. Based on Sichuan, China, an uneven socio-economic development province with plateau, basin, and mountain terrains spanning different climatic zones, we aimed to systematically examine the impacts of floods on diarrheal morbidity.

**Methods:**

We retrieved information on daily diarrheal cases, floods, meteorological variables, and annual socio-economic characteristics for 21 cities in Sichuan from January 1, 2017 to December 31, 2019. We fitted time-series Poisson models to estimate the city-specific floods-diarrhoea relation over the lags of 0-14 days, and then pooled them using meta-analysis for cumulative and lag effects. We further employed meta-regression to explore potential effect modifiers and identify effect modification. We calculated the attributable diarrheal cases and fraction of attributable morbidity within the framework of the distributed lag model.

**Results:**

Floods had a significant cumulative association with diarrhoea at the provincial level, but varied by regions and cities. The effects of the floods appeared on the second day after the floods and lasted for 5 days. Floods-diarrhoea relations were modified by three effect modifiers, with stronger flood effects on diarrhoea found in areas with higher air pressure, lower diurnal temperature range, or warmer temperature. Floods were responsible for advancing a fraction of diarrhoea, corresponding to 0.25% within the study period and 0.48% within the flood season.

**Conclusions:**

The impacts imposed by floods were mainly distributed within the first week. The floods-diarrhoea relations varied by geographic and climatic conditions. The diarrheal burden attributable to floods is currently low in Sichuan, but this figure could increase with the exposure more intensive and the effect modifiers more detrimental in the future. Our findings are expected to provide evidence for the formulation of temporal- and spatial-specific strategies to reduce potential risks of flood-related diarrhoea.

Recently, floods have been wreaking havoc across the world. In mid-July 2021, for example, floods have engulfed several European countries, with more than 170 killed and hundreds missing [1]. In the meantime, Henan province, China, has suffered a flood triggered by record-breaking rainfall, with 12 kills in a flooded subway and hundreds of thousands of evacuations [2]. Floods are usually caused by heavy or frequent rainfall events and have been widely identified as one of the most life-threatening natural disasters around the world [3,4]. Besides mortality, multi-diseases could be induced by floods [5], whose floods-related morbidity risks would raise with the increase in frequency, intensity, and duration of floods in the future [6].

Diarrhoea, a typical disease susceptible to floods, remains a major threat to public health around the world, which ranked as the eighth leading cause of death among all age groups in 2016 [7]. Residents mostly affected by diarrhoea were found to be mainly distributed in underdeveloped regions like Southeast Asia [8,9]. In China, the incidence of diarrhoea ranked the second-highest among notifiable diseases, with a morbidity rate of 92 cases per hundred thousand population reported in 2018 [10].

Growing studies have shed light on the evidence for the association between floods and diarrhoea, especially in China, one of the most flood-prone countries around the world [11]. Despite consensus exists among researchers that floods would increase the morbidity risk of diarrhoea [4,12-16], the available evidence is insufficient to formulate temporal- and spatial-specific interventions. In summary, the evidence on the following questions was believed to remain unclear: a) according to the diarrheal morbidity risk trend after exposure to floods, in what post-flood period should the interventions be performed; b) based on the effect modifiers and their roles, regions with what characteristics should be focused on; c) in terms of the diarrheal morbidity risk attributable to floods, should the flood-induced diarrhoea be a priority in China? Knowledge of the three questions would not only provide a better understanding of the relationship between floods and diarrhoea but provide epidemiological evidence-based implications for planning suitable public health interventions.

At present, however, little is known about the lag effects of floods on diarrheal morbidity as well as the effect modification by different regional, climatic and social characteristics. Furthermore, no study has directly focused on the attributable morbidity risk, neither as absolute excess (numbers) nor relative excess (fractions) of morbidity, which is particularly important to policymakers. Specifically, although a few studies have given the location-specific lag effects of floods on diarrheal morbidity [17,18], most studies based on case-crossover designs [19-23] failed to capture the floods-diarrhoea lag effects, leaving the lag effects far from clear. Meanwhile, several studies have shown the floods-diarrhoea associations varied by location and tried to quantify the effect modification [24-27]. For instance, Thompson et al. found that air temperature and humidity drove spatial heterogeneity in floods-associated diarrheal morbidity risks [21]. However, all these studies had limited between-site variabilities and seldomly considered the effect modifiers (ie, including insufficient effect modification variables), which failed to elaborate on the mechanism of effect modifications. As for attributable morbidity risk, to our best knowledge, only two studies [28,29] used case-crossover designs and the comparative risk assessment framework developed by WHO[30] to calculate the attributable disease burden in terms of years lived with disability (YLDs). Both studies, however, failed to consider any temporal relationship between floods and diarrheal morbidity, which could bias the estimates. Moreover, the two studies provided attributable YLDs instead of attributable morbidity fraction or number of cases, thus did not provide an intuitive sense of flood-related diarrheal risk.

Therefore, we based our analysis on the framework of distributed lag model and aimed to examine: a) the cumulative and lag effects of floods on diarrheal morbidity in 21 cities in Sichuan province, China; b) the effect modification by different climatic areas as well as climatic and social characteristics; c) diarrheal morbidity risks attributable to floods. Our study area, Sichuan, China, an uneven socio-economic development province with plateau, basin, and mountain terrains spanning different climatic zones, enabled us not only to characterize the associations of floods and diarrheal morbidity across various locations with distinct city-specific characteristics but also to explore the effect modifiers and their roles by employing huge spatial heterogeneity of the associations. This setting further enabled us to estimate a more general attributable morbidity risk by taking the huge effect heterogeneity driven by variation in characteristics of flooded cities into account.

## METHODS

### Study area

**Figure 1:**
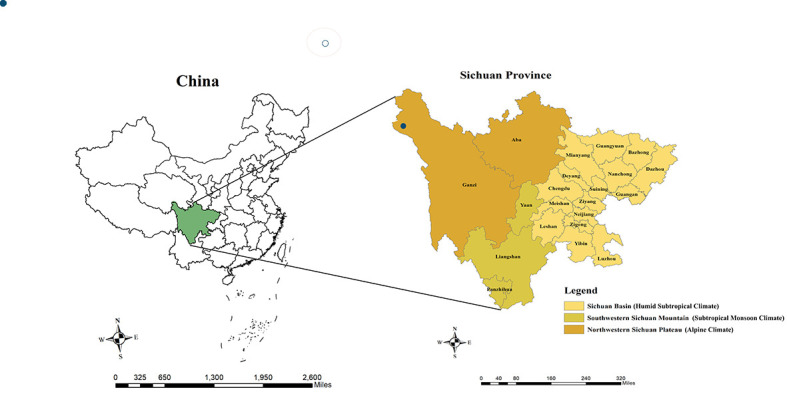
Location of Sichuan province in China and geographic regions in Sichuan province. The blue dots represent the weather stations used in this study.

Our study was based on Sichuan, a southwestern province in China, covering 21 cities in the geographic regions of 97°21′ to 108°33′ east longitude and 26°03′ to 34°19′ north latitude. The GDP per capita and the land area of Sichuan province respectively ranked the nineteenth and the fifth among 31 provinces of Mainland China, with a population of 83.41 million reported in 2018 [31]. The socio-economic development is unevenly distributed across the province (refer to Table S1 in the **Online Supplementary Document** for more details), where eastern Sichuan is generally characterized by a dense population, high-level economic development, and well-developed sanitation infrastructure, while western Sichuan is the opposite situation [32]. The topographic and climatic characteristics across Sichuan are also endowed with huge variations. According to Chengdu Institute of Geography [33] and Sichuan Provincial Meteorological Service [34], the regions of Sichuan can be divided into the Sichuan basin, southwestern Sichuan mountain, and northwestern Sichuan plateau, which respectively correspond to humid subtropical climate, subtropical monsoon climate, and alpine climate (**Figure 1**).

### Data sources

#### Daily diarrheal cases

Information on daily diarrheal cases between January 1, 2017 and December 31, 2019, was retrieved from the Sichuan Center for Disease Control and Prevention. According to the National Health and Family Planning Commission [35], diarrhoea, containing dysentery, cholera, paratyphoid, typhoid, and other infectious diarrhoea, is defined as a group of infectious diseases caused by various pathogens including viruses, bacteria, and parasites, with diarrhoea being the typical symptom. As a notifiable infectious disease in China, all diarrheal cases must be reported via the submission of a standardized online form within 24 hours of diagnosis [36]. In our study, diarrheal cases induced by cholera were not included due to data inaccessibility issues.

#### Floods data

There is no standard floods definition and the most recognized was defined based on yearbooks records [17,37-39]. Based on the Yearbooks of Meteorological Disasters in China, a flood is defined as a natural hazard caused by the overflow of rivers, debris flow, or landslide due to local or regional heavy rainfalls, leading to economic losses and damage to villages, lands, and fatalities. A recorded flood in the Yearbook must fulfil at least one of the following criteria: (1) ten or more people were killed. (2) 50 000 or more hectares of farmland were damaged. (3) the direct economic loss reached more than 100 million CNY. Floods data from 2017 to 2018 were retrieved from the Yearbooks of Meteorological Disasters in China [40,41] which recorded the floods occurrence time and areas. Since the 2020 yearbook was not published yet, floods data in 2019 were retrieved from Sichuan Climate Bulletin [42] which carried the same floods information in Sichuan as the yearbooks.

#### Meteorological data

Daily meteorological data were retrieved from the China Methodological Data Sharing Service System (https://data.cma.cn/). The meteorological variables included the average rainfall, relative humidity, average temperature, maximum temperature, minimum temperature, sunshine duration, and air pressure. The daily diurnal temperature range was further calculated as the difference between the maximum and minimum temperature each day. All missing values (approximately 0.1%) were imputed using the linear interpolation method. For cities with more than one station, we calculated the means of each meteorological variable in each city; for cities with no meteorological monitoring station, relevant data from the station closest to the city centre were used. All the mapped weather stations used in this study were mapped in **Figure 1**, indicated by blue dots.

#### City-specific characteristics

We collected climatic, social, and geographical characteristics for every 21 cities. For each city, we calculated the arithmetic average of the daily meteorological factors (including temperature, relative humidity, sunshine duration, average rainfall, air pressure, and diurnal temperature range) across the study period as an indicator reflective of the city-specific climatic differences. We also calculated the annual wet days for each city as the number of days with one millimetre (mm) or more rainfalls in a calendar year based on the definition of the World Meteorological Organization Commission for Climatology and the Expert Team on Climate Change Detection and Indices [43]. We collected the annual city-specific social characteristics from 2017 to 2019 from the Sichuan Statistical Yearbook [44-46] and calculated the arithmetic average of these annual social characteristics. These social characteristics include socioeconomic variables (population density, GDP per capita, urbanization rate, and traffic) and health resources (hospital beds, licensed physicians, and the number of health institutions). Besides, an indicator variable based on geographic and meteorological division [33,34] was also used to group the 21 cities into three regions mentioned above.

### Statistical analysis

The floods-diarrhoea association was examined with a two-stage statistical model, which has been described in previous papers [47,48]. In summary, we estimated the city-specific floods-diarrhoea association in the first stage. These estimated relations were then pooled in the second stage at provincial and regional levels using meta-analysis.

#### First-stage analysis

In the first stage, we used a time-series quasi-Poisson regression model to obtain city-specific estimates allowing for overdispersion of daily diarrheal counts. Seasonal and long-term temporal trends were controlled for using an indicator with the combination of year, month, and day of the week [18]. Following previous studies [17,49-51], we included daily average relative humidity and temperature, using natural cubic splines with 3 degrees of freedom (*df*), as covariates to control for potential confounding effects. To control for the autocorrelation, the first-order lagged variable of model residuals was incorporated. Taking both the incubation and infectious periods of diarrhoea into account [24,52,53], exposure lagging of up to 14 days was set. To understand the characteristics of lag effects of floods on diarrhoea, a natural cubic spline with 4 *df* was used to capture the distributed lag effect over time up to 14 days. The following statistical formula was applied to each city to obtain the city-specific estimates.

*Log* [*E*(*Y_t_*)] = *cb* (*Floods_t_*) + *ns* (*Hum_t_*, *df* = 3) + *ns* (*Tem_t_*, *df* = 3) + *Strata* + *Lag* (*res*, 1)

Where *Y_t_* represented the number of daily cases of diarrhoea on day *t*. *Floods_t_*, a categorical variable indicating flood occurred on day *t* (coded as 1) or not (coded as 0), was applied with the cross-basis function (*cb*) where a linear relationship (*df* = 1) for exposure-response dimension and a natural cubic spline with 4 *df* for lag-response dimension were set. *Hum_t_* and *Tem_t_* were the daily average relative humidity and temperature. *ns* represented natural cubic spline. *Strata* was an indicator with the combination of year, month, and day of the week. *Lag* (*res,*1) was the first-order lagged variable of model residuals.

#### Second-stage analysis

In the second stage analysis, the univariate meta-analyses with random intercepts [47,48] were used to pool the city-specific cumulative estimates of floods (lag 0-14 days) obtained from the first-stage models to obtain province and region-level pooled estimates.

A multivariate meta-analysis with a random intercept was employed to pool the distributed lag terms expressing the lag-response relations specific to the flood days compared to the non-flood days. This method was described previously [48].

The floods-diarrhoea associations were expressed as the relative risks (RRs) and 95% confidence intervals (CIs) of diarrheal cases associated with flood days in comparison to non-flood days.

#### Exploring the potential effect modifiers

Employing the spatial heterogeneity of the city-specific cumulative effect estimates obtained from the second-stage analysis, meta-regression models were used to further explore the potential effect modifiers. We incorporated each city-specific characteristic separately as the meta-predictor into the meta-regression model, where the city-specific cumulative effect estimates (lag 0-14 days) were the values of the dependent variable. Due to the limited sample size (21) embedded in the meta-regression, we categorized these meta-predictors into high- and low-groups by taking provincial median values as cut-points. We employed the likelihood ratio (LR) test to examine the statistical significance of each meta-predictor.

#### Diarrhoea risk attributable to floods

To account for the potential effect modification, we incorporated the statistically significant meta-predictors obtained from the last step into a meta-regression model. We then used the fitted meta-regression model to derive the best linear unbiased prediction (BLUP) of the cumulative exposure-response association (lag 0-14 days) in each city, the benefits of which were discussed previously [54]. We estimated the attributable number of diarrheal cases and the fraction of attributable cases using a newly proposed method [55], which overweighs the traditional methodology by considering the lag effect in the context of distributed lag models.

The attributable risks for the whole study period and the flood season (every May to October) were estimated. Based on the attributable risk during flood season, we further calculated the subgroup attributable risks by effect modifiers. Technical details can be seen in Text A1 in the **Online Supplementary Document**.

#### Sensitivity analysis

Residual analyses and partial autocorrelation figures were first performed to evaluate the developed models in the first-stage analysis. We further examined the sensitivity of the main findings with respect to: (1) Varying the *df* (2-5) for the covariates of daily relative humidity and temperature. (2) Varying the *df* (2-5) for the lag-response dimension of the cross-basis function.

All statistical analyses were conducted with R software (version 4.0.3, developed by R Core Team) (Foundation for Statistical Computing, Vienna, Austria), mainly employing the packages *dlnm* and *mvmeata*. The geographic map was made by ArcGIS software (version 10.0 developed by Esri, Redlands, USA, authorization number: EFL734321752).

## RESULTS

### Descriptive statistics for the diarrhoea disease and floods

**Figure 2:**
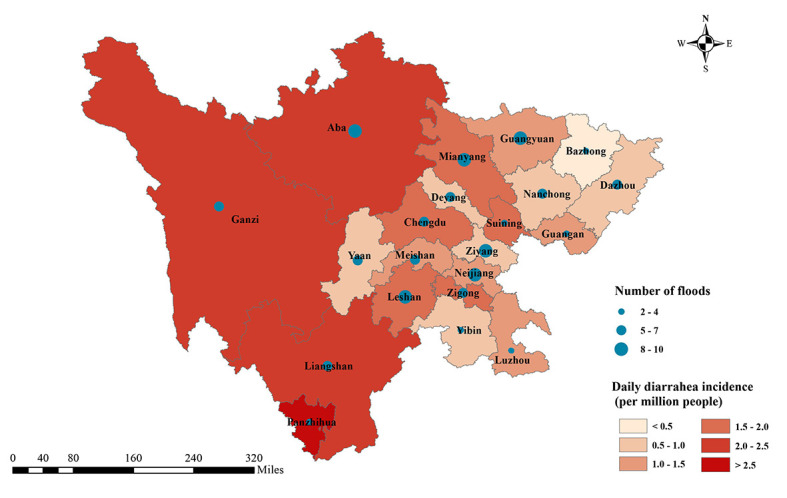
Summary of the total number of floods and average daily diarrheal incidence (per million people) in each city of Sichuan province, from January 2017 to December 2019. The average daily diarrheal incidence for each city was calculated as the total number of diarrheal cases per million people divided by the total number of days during the study period.

This study covered 21 cities in Sichuan province, China, with a total of 124 602 diarrheal cases identified among more than 86 million people from January 1, 2017 to December 31, 2019. **Figure 2** shows the summary of the total number of floods and the average daily diarrheal incidence (per million people) in each city of Sichuan province across the study period. The number of floods varied across cities, resulting in different flooding days for cities. Table S1 in the **Online Supplementary Document** provides more detailed descriptive statistics from each city.

**Figure 3:**
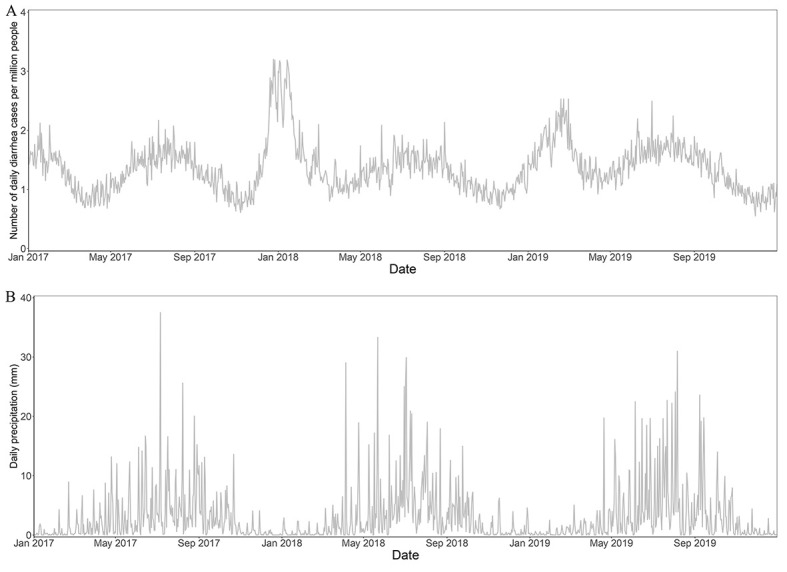
Daily diarrheal cases per million people (**Panel A**) and daily precipitation (**Panel B**) in Sichuan province of China, from January 2017 to December 2019.

**Figure 3** demonstrates the number of daily diarrheal cases per million people (**Figure 3**, Panel A) and daily precipitation (**Figure 3**, Panel B) in Sichuan, China. Compared to the dry season, the lower daily diarrheal incidence during the flood season (every May to October in Sichuan) hints that flood is not a dominating risk factor for diarrhoea, implying that the morbidity risk attributable to floods may not be high.

The summary statistics of the city-specific characteristics (meta-predictors) were listed in the **Online Supplementary Document**, which showed significant variations across cities (see Table S2 in the **Online Supplementary Document**).

### Association between floods and diarrhoea

**Figure 4:**
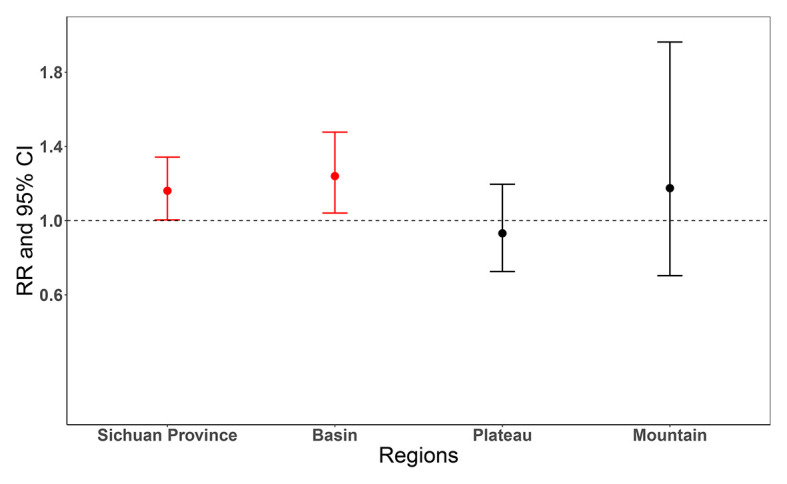
Cumulative effects of floods on diarrheal morbidity over lag 0-14 days in Sichuan province and 3 climatic areas. Please refer to **Figure 1** for climatic areas definitions. RR = relative risk, CI = confidence interval.

**Figure 5:**
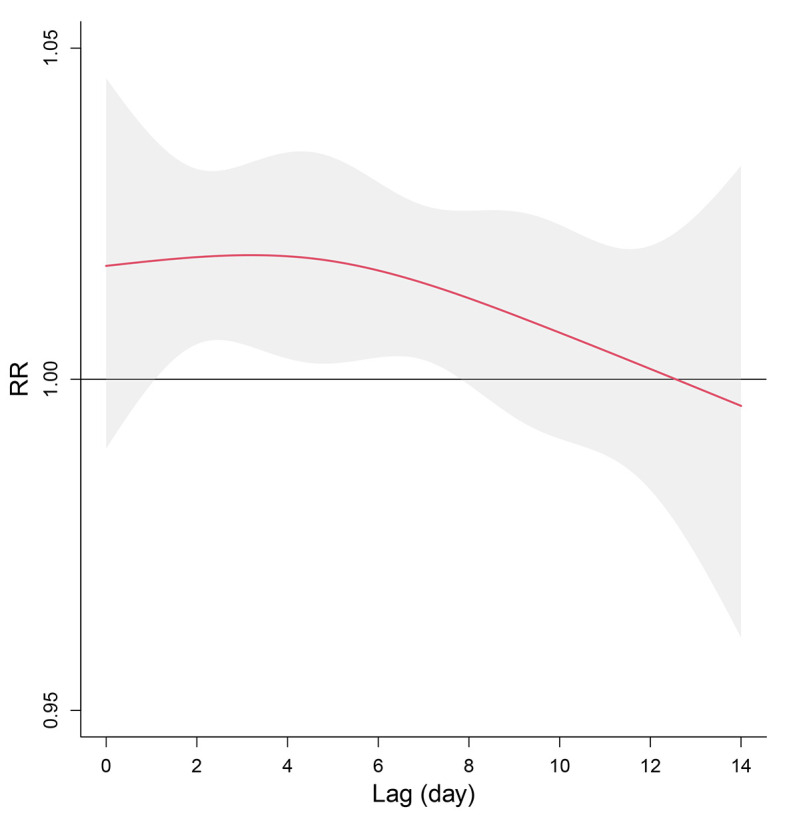
Lag effects of floods on diarrheal morbidity along with lag 0-14 days in Sichuan province.

**Figure 4** illustrates the overall cumulative effects of floods on diarrheal morbidity in Sichuan province and 3 climatic areas. The corresponding results for all 21 cities were reported in Table S3 in the **Online Supplementary Document**. At the provincial level, floods were demonstrated to be significantly associated with diarrheal cases with a RR equalling 1.16 (95% CI = 1.004-1.342). At finer scales, the cumulative effects were positively statistically significant in the Sichuan basin (humid subtropical climate), with no statistical significance identified in the other two regions. As shown in **Figure 5**, the estimated effects of floods on diarrheal morbidity in Sichuan province appeared on the second day after the flood and lasted for 5 days. Figure S1 in the **Online Supplementary Document** provides the details for the city-specific lag effect of floods on diarrheal morbidity.

**Figure 6:**
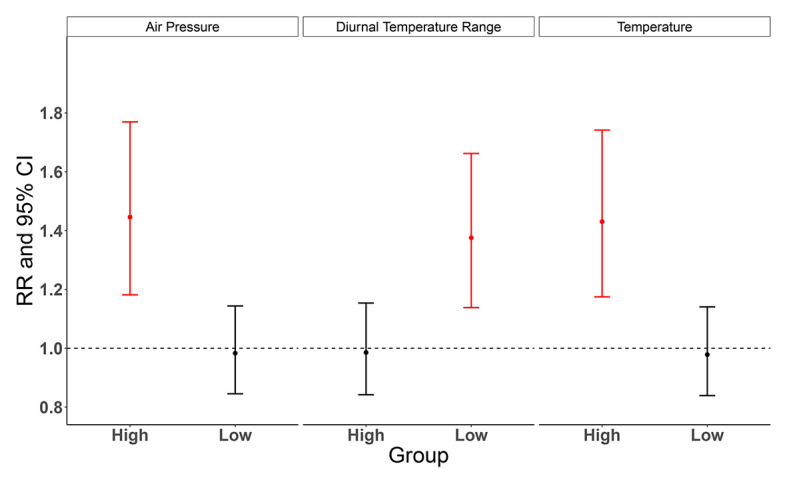
Modification effect of meta-predictors on the cumulative effects of floods on diarrheal morbidity. RR represents the cumulative effect of floods on diarrheal morbidity, derived from the univariate meta-regression model, and CI represent confidence interval. Meta-predictors were categorized into the high and low groups by taking provincial median values as cut-points, and the high group was taken as a reference group.

Results from univariate meta-regression models show that the city-specific characteristics of air pressure, diurnal temperature range and temperature can modify the cumulative effect of floods on diarrheal morbidity (Table S4 in the **Online Supplementary Document**). The cumulative effects of floods on diarrheal morbidity were found to be significantly stronger in regions with higher air pressure, lower diurnal temperature range, or warmer temperatures (**Figure 6**).

### Attributable risk

**Table 1** shows the attributable morbidity risks and 95% empirical CIs. Overall, the total number of attributable diarrheal cases in Sichuan was 310 (95% eCI = 123-454) which resulted in the total attributable fraction of 0.25% (95% eCI = 0.10-0.36) and that of flood season (every May to October during the study period) 0.48% (95% eCI = 0.19%-0.71%). During flood season, the attributable fractions in areas with high temperature, high air pressure, and low diurnal temperature range were estimated to be 1.45%, 1.47%, and 1.41% respectively, with all estimates found to be statistically significant. The attributable risks varied substantially between cities (Table S5 in the **Online Supplementary Document**). Statistically insignificant attributable fractions were found in several cities, which can be caused by the low frequency of exposure to floods or different floods-diarrhoea patterns.

**Table 1:** Attributable morbidity risk and 95% empirical CI*

Group	Total diarrheal cases	Attributable diarrheal cases	Attributable fraction
Whole study period	124 602	310 (123-454)	0.25% (0.10-0.36)
Flood season period†	64 124	310 (123-454)	0.48% (0.19-0.71)
Temperature: high	24841	360 (276-420)	1.45% (1.11-1.69)
Temperature: low	39 283	-50 (-220, 88)	-0.13% (-0.56, 0.22)
Air pressure: high	24 653	362 (278-423)	1.47% (1.13-1.71)
Air pressure: low	39 471	-52 (-225, 84)	-0.13% (-0.57, 0.21)
Diurnal temperature range: high	38 719	-49 (-222, 82)	-0.13% (-0.57, 0.21)
Diurnal temperature range: low	25 405	358 (257-431)	1.41% (1.01-1.70)

CI – confidence interval

*The subgroup attributable risks by effect modifiers were estimated based on attributable risk during flood season.

†Flood season represents every May to October during the study period.

### Sensitivity analysis

Figure S2 in the **Online Supplementary Document** shows that residuals of the developed models in the first-stage analysis were randomly distributed, and Figure S3 in the **Online Supplementary Document** implies there was no obvious autocorrelation of residuals in most of these models. Our results were robust to modified degrees of freedom (2-5 *df*) for the daily relative humidity, daily temperature, and lag-response dimension of the cross-basis function. (see Figure S4, Figure S5, and Table S6 in the **Online Supplementary Document**).

## DISCUSSION

Our findings show that floods were associated with an increased risk of diarrheal morbidity at the provincial level, but the estimated effects varied by regions, with a positive association identified in Sichuan Basin (humid subtropical climate) while no statistically significant effects in the other two regions. In general, the lag effects appeared on the second day after exposure to flood and lasted for 5 days. The effects of floods were modified by three effect modifiers, with stronger effects found in areas with higher air pressure, lower diurnal temperature range, or warmer temperature. We found that floods were responsible for advancing a fraction of diarrheal morbidity risk, corresponding to 0.25% in Sichuan within the study period and 0.48% within the flood season (May to October).

Our findings that floods had statistically significant impacts on diarrhoea have been strongly indicated by the ensemble of previous studies [16,56]. Several plausible mechanisms have been proposed for the positive relation. On the one hand, floods could significantly affect the living environment and facilitate the growth and reproduction of pathogens like *E.coli* [57]. On the other hand, floods could increase the risks of human exposure to diarrhoea pathogens by flushing pathogens from environmental reservoirs or faecal matter into waterways used as the daily source of drinking water [58-61]. Therefore, the risks of diarrheal morbidity are thought to be increased by floods from the perspective of pathogens’ biophysics, hygiene conditions, and residents’ behaviours.

Our results also suggest that there was substantial spatial heterogeneity in the floods estimates. In particular, the statistically significant effect was only identified in the Sichuan basin (humid subtropical climate). The potential reason might be that the characteristics of floods in the Sichuan basin are different from the other two regions. As indicated by Zhang et al. [62], compared with the other two regions, the Sichuan basin is more prone to suffer debris flow and landslides caused by heavy rainfalls. Therefore, for the Sichuan basin that is typically surrounded by rivers, heavy rainfalls are more likely to simultaneously cause an overflow of rivers, debris flow, and landslide, resulting in greater flood intensity.

Understanding the lag pattern of floods on diarrhoea helps formulate temporal-specific interventions. We based our analysis on the framework of the distributed lag model to characterize the lag-response curve. Our finding suggests that the effect of floods appeared on the second day after the flood and lasted for 5 days, which is approximately consistent with the findings from previous studies [17,18] that the impacts imposed by floods were distributed within the first week. This implies that public health interventions should be promptly performed after floods to reduce floods-related diarrheal morbidity.

Determining the effect modifiers and their roles is conducive to shedding light on regions with what characteristics should be focused on and further formulating spatial-specific interventions. Our study identified three effect modifiers, with stronger flood effects on diarrheal morbidity found in areas with higher air pressure, lower diurnal temperature range, or warmer temperature. Our previous study focusing on hand, foot, and mouth disease reported that a lower diurnal temperature range could increase the risk of disease [63]. The underlying reason for this was that a small diurnal temperature range usually combines high humidity and short sunshine hours, which could be beneficial for the survival of pathogens and the prevalence of waterborne disease [64-66]. For air pressure, the strong negative association between air pressure and diurnal temperature range might serve as a potential explanation for our results. Meanwhile, warmer temperatures may cause increased pathogen proliferation in food and drinking water [60,67], leading to an increase in the diarrheal morbidity risk. However, it should be noted that it would be premature to consider effect modifiers serve as predictors of diarrheal morbidity since we only explore their effect modification in this study.

An interesting finding is that although the overall diarrheal morbidity fraction attributable to floods is statistically significant, the estimate is small (0.25% within the study period and 0.48% within the flood season). Comparison with these previous studies like [28,29] is limited by several factors, particularly the alternative definitions of attributable risk measures and the variations in study designs and modelling approaches. Nonetheless, we can determine the generalizability of our results by anatomizing the estimation process of attributable risk. Specifically, both the intensity of floods’ effect on diarrheal morbidity and the frequency of exposure to floods would contribute to the estimates (please refer to Text A1 for technical details). On the one hand, the RR in Sichuan (1.16 in this study) is relatively low compared with the previously reported RR in other regions of China (around 1.2) [17,68,69]. This could be explained by the well-developed sanitation infrastructure [70] and distinct climatic characteristics [41] in Sichuan. On the other hand, the frequency of exposure to floods in Sichuan is relatively low compared with areas on the eastern coast and some regions in the middle plain surrounded by large rivers [71]. Therefore, the diarrheal morbidity risk attributable to floods in the whole of China could be higher than our estimate. It would be premature to consider floods an unimportant risk factor for diarrhoea, even we presume the attributable diarrheal morbidity risk is currently modest in the whole of China. In the context of climate change, the continuing decrease in the diurnal temperature range [72] and increase in temperature [73] would increase the risks of flood-related diarrheal morbidity. Besides, the frequency, intensity, and duration of floods will significantly increase in the future [6], which has the potential to greatly exacerbate the diarrheal morbidity risk attributable to floods. Therefore, floods-related diarrhoea remains a concern in China.

Several limitations should be noted in this study. The first pertains to the intrinsic nature of ecological studies. Specifically, the analysis in this study established a correlation rather than causality thus our results have limitations in causal inference. Second, the diarrheal cases in the study remain under-reported as individuals with mild clinical symptoms might not choose to visit physicians, thus leading to an underestimation of the impact of floods. Third, the cases of cholera were not included in our study due to the inaccessibility of the related data, leading to an underestimation of the impact of floods. However, given the rare incidence of cholera cases among general populations, we do not think this would make a big difference to our results. Fourth, our data set did not include the characteristics of floods such as the type and intensity, so we investigated all floods recorded in the Yearbooks of Meteorological Disasters in China. Last, the study area in this study was limited to Sichuan Province, whose results and subsequent conclusions should be prudent to be generalized to the nationwide.

Despite all the limitations mentioned above, our study demonstrated a major strength that our systematic analysis of data from 21 cities in Sichuan province provided evidence for the floods-diarrhoea relationship in a wide range of climates and regions with distinct socio-economic and infrastructural characteristics. Besides, previous studies in China did not capture the effect of floods in regions with plateau terrain [17,18,68]. Our study bridged this gap by reporting the absence of statistically significant effects in plateau and alpine climatic regions. Our results also provide political implications for the formulation of temporal- and spatial-specific interventions by determining when and where the public health interventions should be performed and whether flood-induced diarrhoea should be a priority in China.

## CONCLUSIONS

We identified a statistically significant effect of floods on diarrheal morbidity, with estimates that varied by regions. The effects appeared on the second day after exposure to the flood and lasted for 5 days. The floods-diarrhoea associations were modified by three effect modifiers, with stronger flood effects on diarrheal morbidity found in areas with higher air pressure, lower diurnal temperature range, or warmer temperature. These findings imply that public health interventions should be promptly performed after exposure to floods, especially for areas with low altitude, small diurnal temperature range, or warmer temperatures. Our study also suggests that floods were responsible for advancing a fraction of diarrheal morbidity. Although the attributable risk associated with floods is currently small in Sichuan, it would be premature to consider floods an unimportant risk factor for diarrhoea, as this figure could be higher in the whole of China and could significantly increase with the enhanced floods-diarrhoea association and more dangerous exposure in the future. Overall, our study is expected to provide a better understanding of the relationship between floods and diarrheal morbidity, which would further provide epidemiological evidence-based implications for policy-making procedures aimed at reducing the potential risks of flood-related diarrhoea via the adoption of effective temporal- and spatial-specific strategies.

## Additional material


Online Supplementary Document
