# Supplementary material for: Floods and diarrheal morbidity: Evidence on the relationship, effect modifiers, and attributable risk from Sichuan Province, China
Source: J Glob Health. 2022 Jul 25;12:11007. doi: 10.7189/jogh.12.11007 (PMC9308977; doi:10.7189/jogh.12.11007)
Supplement: Online Supplementary Document [file jogh-12-11007-s001.pdf]

Online Supplementary Document

Text A1: Details on the computation of the attributable risk

The diarrhea risk attributable to a flood indicator $x_{i,t}$ in a city i for a given day t is defined as the number $AN_{x,i,t}$ and fraction $AF_{x,i,t}$ of morbidity experienced in the next L days, with L as the maximum lag period (14 days in our study), defined by:

$$AF_{x,i,t} = 1 - \exp(-\sum_{l=0}^{L} \beta_{x_{i,t},l})$$

$$AN_{x,i,t} = AF_{x,i,t} \cdot \sum_{l=0}^{L} \frac{n_{i,t+l}}{L+1}$$

where $\sum \beta_{x_{i,t},l}$ is the overall cumulative log-relative risk for flood indicator $x_{i,t}$ in city i and day t, and $n_{i,t}$ is the number of diarrheal cases in city i and day t. The city-specific risk estimate $\sum \beta_{x_{i,t},l}$ is obtained by the BLUP of the overall cumulative exposure-response association specific to the flood compared to the non-flood. The non-flood is therefore the counterfactual condition for the definition of the attributable risk. Thus, the attributable risk can be interpreted as the excess morbidity due to floods, if compared to a hypothetical situation in which no flood occurs during the study period.

The city-specific total attributable number of diarrheal cases AN_{tot_i} due to floods is given by the sum of $AN_{x,i,t}$ in a city i for all the days t of the study period, and its ratio with the total number of diarrheal cases in the city provides the city-specific total attributable fraction AF_{tot_i}. The provincial total attributable number of diarrheal cases AN_{tot} is given by the sum of AN_{tot_i} for all cities i of

Sichuan province, and its ratio with the total number of diarrheal cases in Sichuan provides the provincial total attributable fraction AF_{tot}. The attributable fraction of flood season $AF_{flood\ season}$ (every May to October during the study period) is further calculated as the AN_{tot} divided by the number of diarrheal cases during the flood season. The subgroup attributable cases by modifiers are further calculated as the sum of $AN_{tot_{i:i \in modifier\ subgroups}}$, and its ratio with the total number of diarrheal cases during the flood season provides the subgroup attributable fraction by modifiers $AF_{modifier\ subgroups}$.

We calculated empirical CIs (eCIs) using Monte Carlo simulations, assuming a multivariate normal distribution of the of the reduced coefficients.

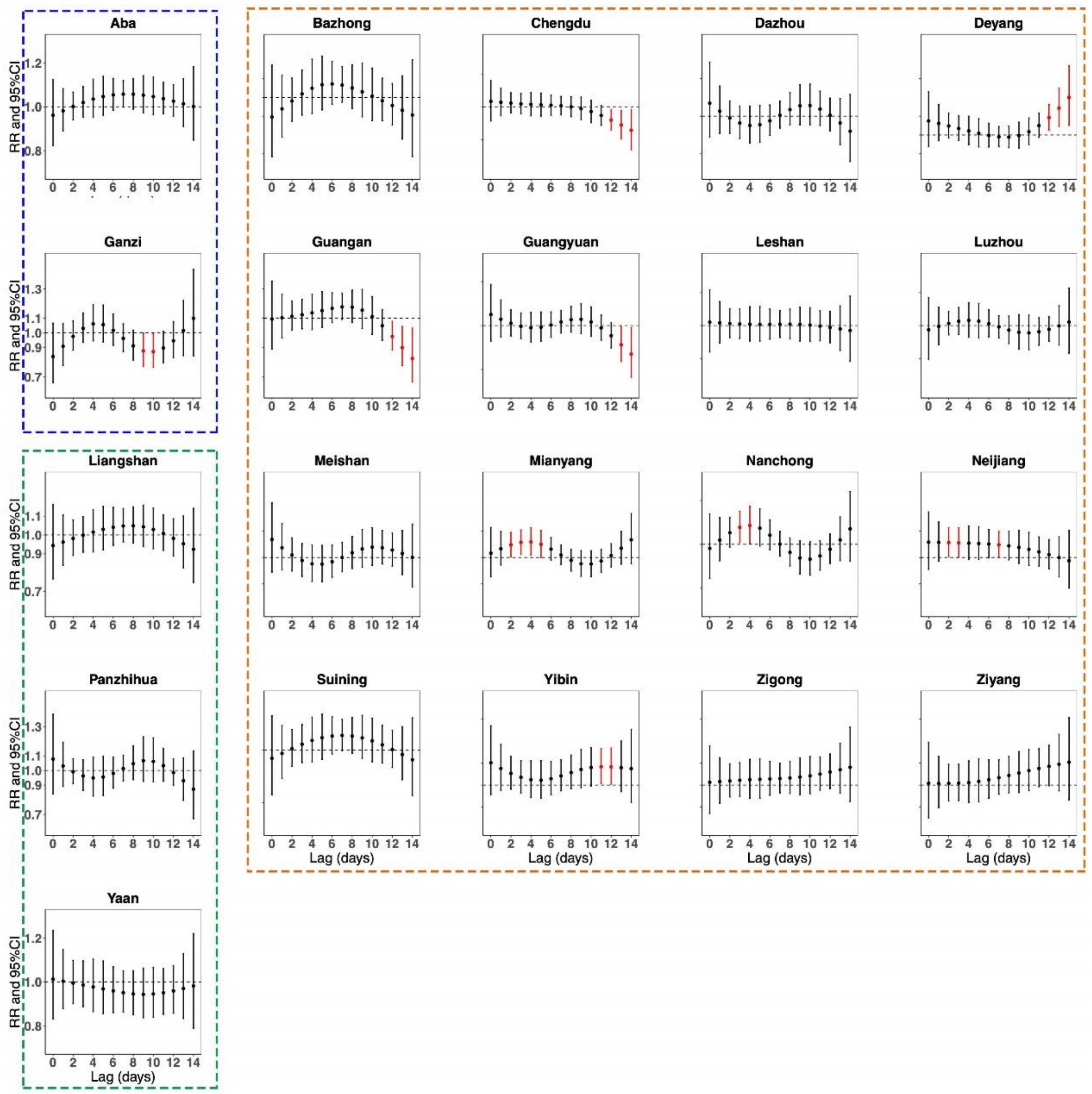

Figure S1: City-specific RR estimates of the flood on the risk of diarrhea along lag 0-14 days in Sichuan province, China. The red lines indicate RR with $P < 0.05$. The cities within blue, green and orange border lines represent northwestern Sichuan plateau (alpine climate), southwestern Sichuan mountain (subtropical monsoon climate), and Sichuan basin (humid subtropical climate), respectively.

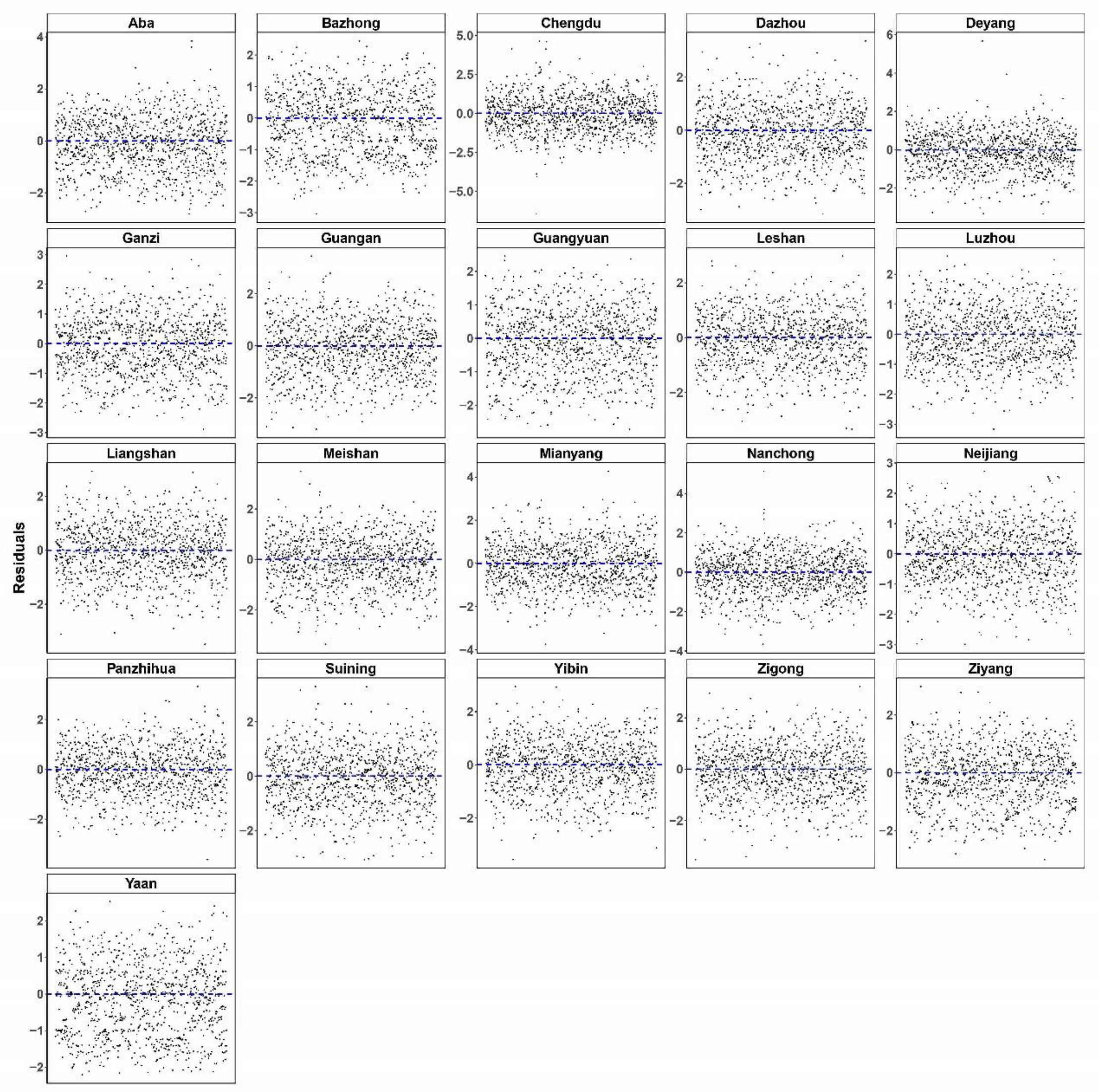

Figure S2: Goodness-of-fit of DLNM models for all 21 cities in Sichuan province. The dotted line shows the fitted values and the ordinate shows the residuals.

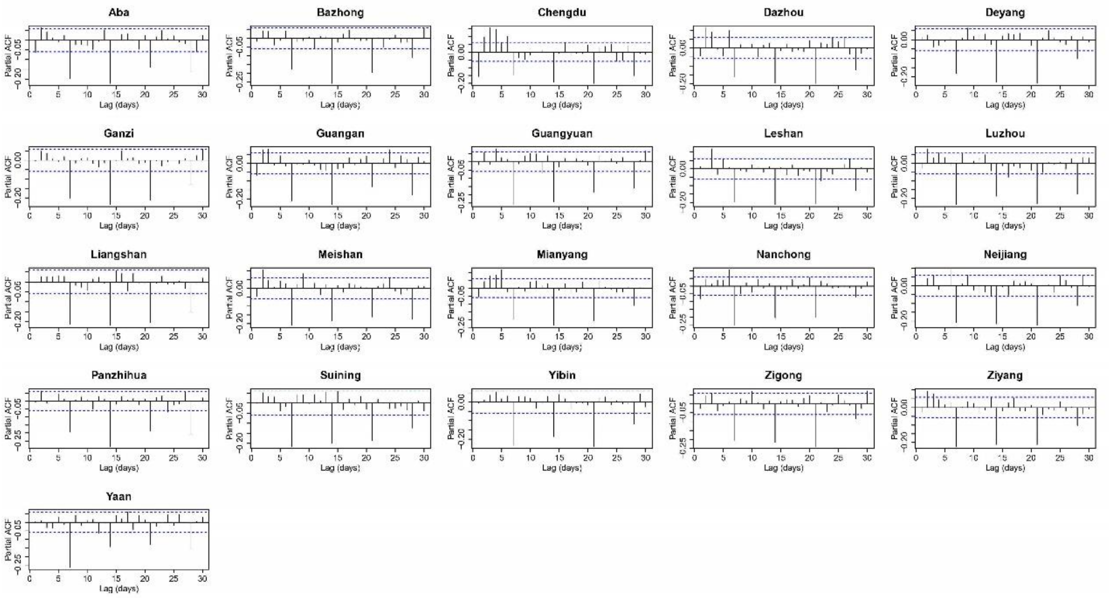

Figure S3: Partial auto-correlation function plots for residuals of the DLNM models.

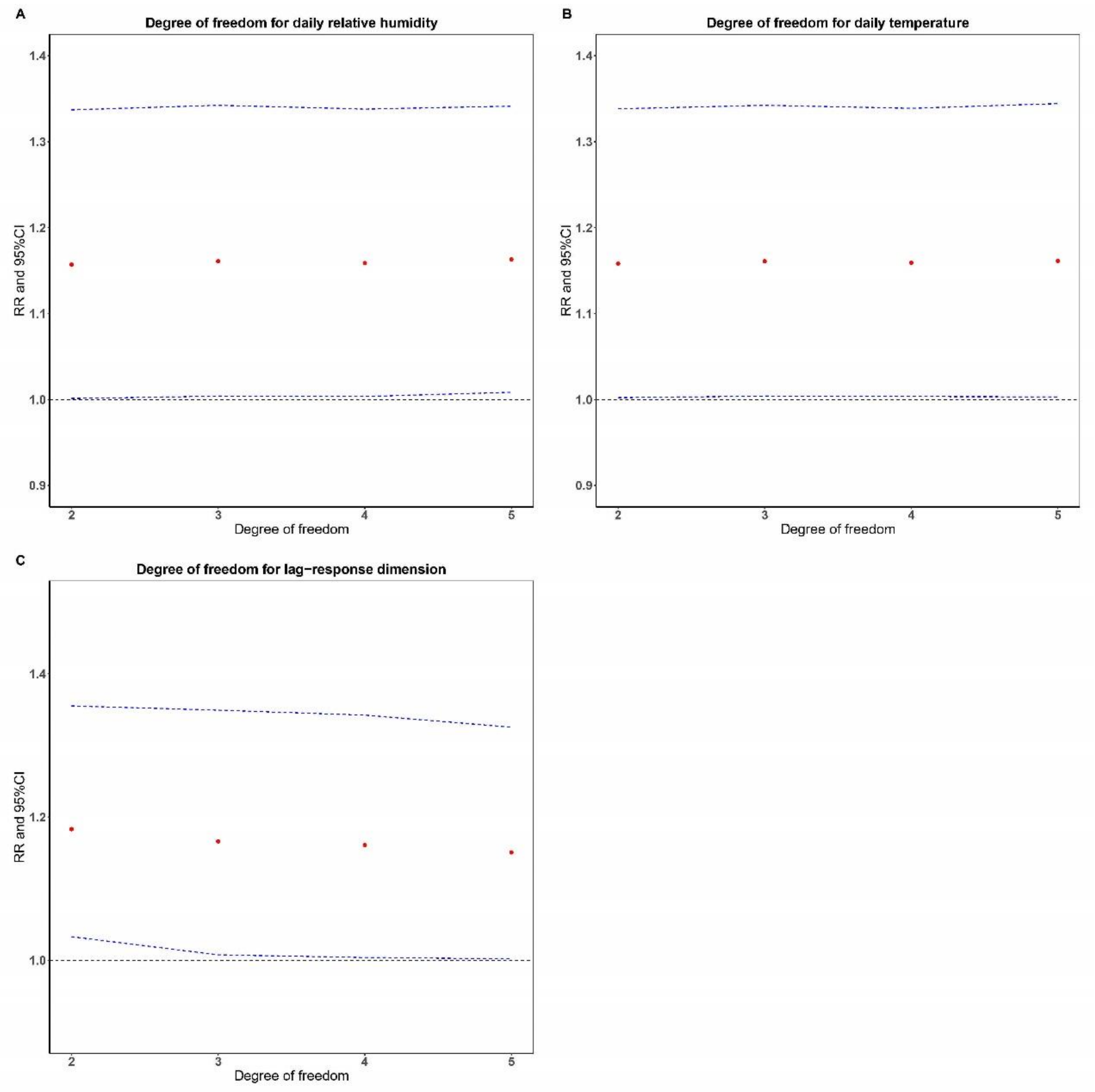

Figure S4: Sensitivity analysis for provincial cumulative effect of floods on diarrhea over lag 0-14 days.

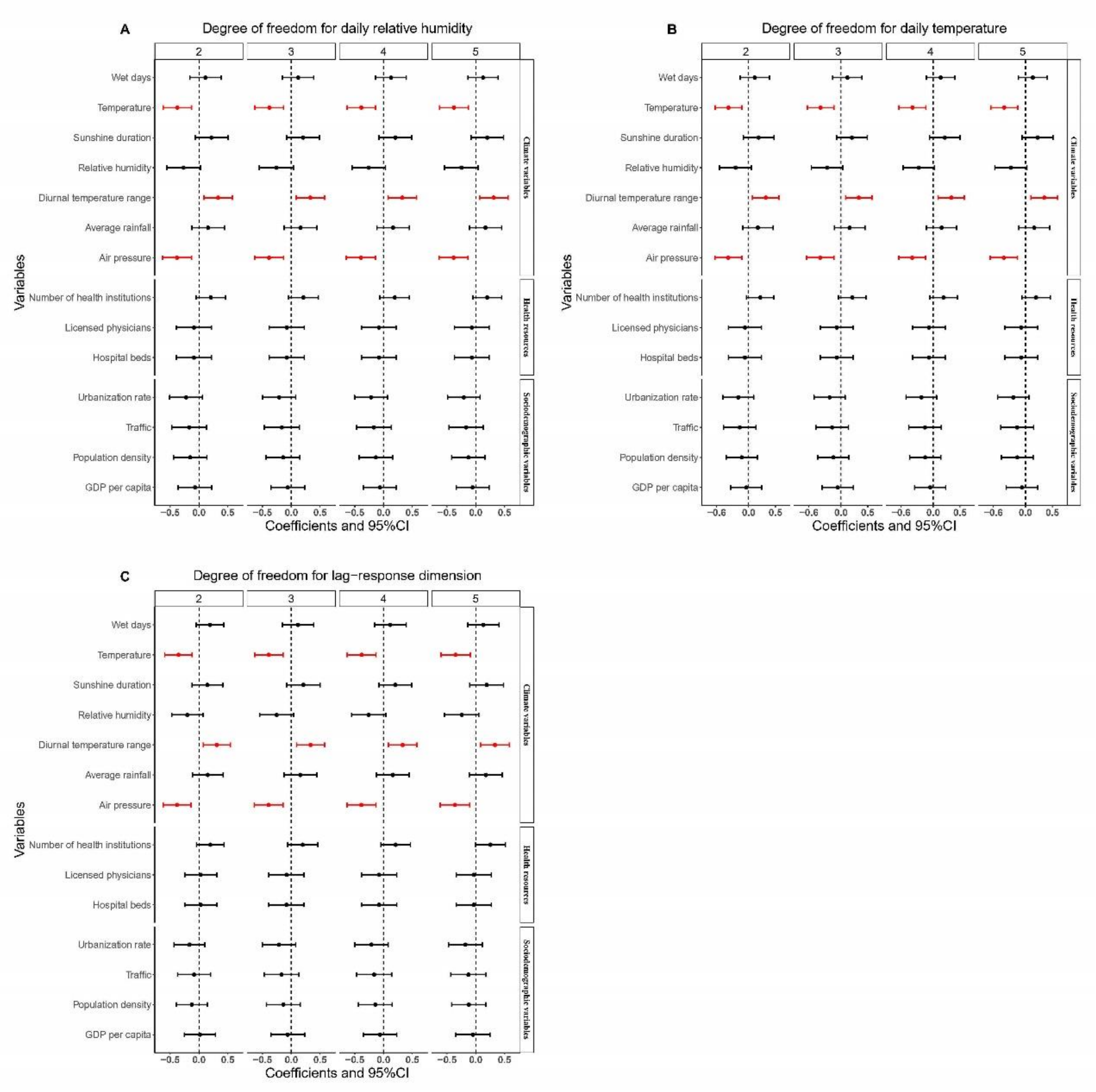

Figure S5: Sensitivity analysis for meta-regression models.

Table S1: Descriptive statistics by city, from January 2017 to December 2019.

Region	City	Average population (million)	Average daily diarrheal cases	Average daily diarrheal incidence [a]	Total number of flooding days	Total number of floods
Sichuan Basin (Humid Subtropical Climate)	Bazhong	3.32	1.40	0.42	21	4
	Chengdu	16.33	29.77	1.82	24	6
	Dazhou	5.72	3.77	0.66	25	6
	Deyang	3.55	3.49	0.98	31	6
	Guangan	3.24	4.16	1.28	18	3
	Guangyuan	2.67	3.08	1.16	40	10
	Leshan	3.27	5.33	1.63	29	9
	Luzhou	4.32	4.95	1.14	9	2
	Meishan	2.98	4.01	1.34	23	6
	Mianyang	4.86	8.18	1.68	39	9
	Nanchong	6.44	3.77	0.59	29	7
	Neijiang	3.70	5.12	1.38	31	9
	Suining	3.20	5.06	1.58	21	4
	Yibin	4.56	4.09	0.90	17	4
	Zigong	2.92	4.46	1.53	18	5
	Ziyang	2.51	2.19	0.87	28	8
Southwestern Sichuan Mountain (Subtropical Monsoon Climate)	Liangshan	4.91	11.47	2.34	23	6
	Panzhihua	1.24	3.24	2.62	16	3
	Yaan	1.54	1.31	0.85	22	7
Northwestern Sichuan Plateau (Alpine Climate)	Aba	0.94	2.29	2.43	32	9
	Ganzi	1.20	2.67	2.23	10	6

[a] Average daily diarrheal incidence (per million people).

Table S2: Description of meta-predictors for the 21 cities in Sichuan, 2017-2019

City	Temperature (°C)	Relative humidity (%)	Sunshine duration (hours)	Average rainfall (mm)	Air pressure (hPa)	Wet days (days)	Diurnal temperature range (°C)	Population density (person/sq. km)	GDP per capita (1000)	Urbanization rate (%)	Traffic[a]	Hospital beds (1000)	Licensed physician (1000)
Sichuan Basin (Humid Subtropical Climate)													
Bazhong	17.17	76.67	4.03	3.79	960.84	102	7.46	270	20.11	41.91	1.72	13.14	10.29
Chengdu	16.36	81.33	2.73	3.37	943.06	124	7.72	1138.33	95.03	73.13	9.12	114.30	101.53
Dazhou	16.83	75.91	3.55	3.59	956.14	108	7.92	344.67	31.11	45.53	2.77	19.88	15.56
Deyang	16.21	80.23	2.49	3.71	934.89	123	7.36	600.33	61.31	52.41	2.39	16.03	14.28
Guangan	17.46	79.19	3.50	3.13	973.68	104	7.06	512.33	37.69	41.79	0.99	13.27	9.36
Guangyuan	16.16	70.02	3.59	2.87	952.01	92	9.06	163.67	31.01	45.60	1.02	15.60	10.46
Leshan	11.14	79.89	3.18	4.48	834.07	160	6.99	257	50.84	51.79	1.49	16.88	12.42
Luzhou	18.79	80.56	3.18	3.01	969.75	139	7.63	353.33	41.45	50.47	3.67	21.23	16.55
Meishan	17.84	80.19	3.79	2.79	965.61	90	7.81	418	42.64	46.31	1.24	12.82	10.14
Mianyang	17.86	75.72	3.29	2.91	970.01	87	7.50	240	49.75	52.56	2.29	24.79	19.85
Nanchong	17.66	77.45	3.39	3.02	971.84	106	7.28	515.33	31.93	48.11	3.13	30.43	20.92
Neijiang	17.98	81.85	3.12	2.42	973.19	94	7.37	690.33	37.38	49.19	4.12	16.80	12.06
Suining	17.78	81.53	3.06	2.53	972.78	95	7.42	603	38.30	50.02	1.28	13.74	10.59
Yibin	18.12	81.68	2.96	2.86	962.13	122	6.77	343.33	47.49	49.65	1.83	24.61	15.87
Zigong	17.98	81.85	3.12	2.42	973.19	94	7.37	665.33	47.81	52.54	1.19	16.03	11.40
Ziyang	17.84	80.19	3.79	2.79	965.61	90	7.81	439.33	37.76	42.73	1.21	11.56	8.50
Southwestern Sichuan Mountain (Subtropical Monsoon Climate)													
Liangshan	14.62	66.66	5.42	2.65	810.43	139	11.06	81	32.08	35.71	3.39	19.33	14.17

Panzhihua	21.26	55.84	6.91	2.21	874.38	67	12.36	166	89.99	66.45	0.67	9.20	6.24
Yaan	16.34	69.22	3.39	2.07	890.57	95	7.53	102	42.71	46.86	0.68	10.44	6.98
Northwestern Sichuan Plateau (Alpine Climate)													
Aba	6.45	64.19	5.55	2.31	705.55	155	14.25	11	35.11	40.02	1.77	3.51	3.7
Ganzi	7.67	54.74	5.76	1.92	687.14	157	14.06	8	26.33	31.72	1.51	3.46	3.39

[a] Traffic represents passenger-kilometers of highways (billion passenger-km).

Table S3: City-specific cumulative effects of floods on diarrhea over lag 0-14 days [a]

City	RR (95% CI)
Sichuan Basin (Humid Subtropical Climate)	
Bazhong	0.92 (0.32, 2.66)
Chengdu	0.86 (0.64, 1.16)
Dazhou	0.99 (0.42, 2.34)
Deyang	**1.99 (1.08, 3.65)**
Guangan	0.72 (0.27, 1.9)
Guangyuan	0.88 (0.5, 1.53)
Leshan	1.02 (0.64, 1.62)
Luzhou	1.00 (0.37, 2.65)
Meishan	1.22 (0.7, 2.14)
Mianyang	1.40 (0.91, 2.14)
Nanchong	1.18 (0.61, 2.28)
Neijiang	**1.74 (1.05, 2.88)**
Suining	1.30 (0.65, 2.62)
Yibin	**2.42 (1.23, 4.76)**
Ziyang	1.86 (0.84, 4.16)
Zigong	1.72 (0.79, 3.72)
Southwestern Sichuan Mountain (Subtropical Monsoon Climate)	
Liangshan	0.96 (0.72, 1.27)
Panzhihua	0.93 (0.48, 1.8)
Yaan	0.63 (0.22, 1.8)
Northwestern Sichuan Plateau (Alpine Climate)	
Aba	1.46 (0.82, 2.61)
Ganzi	0.55 (0.19, 1.64)

[a] Number in bold indicate $P < 0.05$.

Table S4: Coefficients from the meta-regression models

Meta-predictors	β [a]	LR test [b]
Climatic variables[c]		
Temperature	-0.38	0.005
Relative humidity	-0.26	0.086
Sunshine duration	0.21	0.162
Average rainfall	0.16	0.266
Air pressure	-0.39	0.005
Wet days	0.12	0.457
Diurnal temperature range	0.33	0.014
Sociodemographic variables [c]		
Population density	-0.14	0.346
GDP per capita	-0.06	0.686
Urbanization rate	-0.21	0.153
Traffic	-0.16	0.297
Health resources		
Hospital beds	-0.08	0.628
Licensed physicians	-0.08	0.628
Number of health institutions	0.21	0.168
Regions		
Northwestern Sichuan Plateau (Ref.)		
Sichuan Basin	0.83	0.280
Southwestern Sichuan Mountain	0.48	

[a] β is the coefficient of each meta-predictor, which was obtained using the restricted maximum-likelihood method.
[b] Data are *P* values
[c] Variables in each category were categorized into high and low group by taking provincial median values as cut-points,
and high group was taken as a reference group.

Table S5: City-specific attributable morbidity risk and 95% eCI [a]

City	Total diarrheal cases	Attributable diarrheal cases	Attributable fraction
Sichuan Basin (Humid Subtropical Climate)			
Bazhong	1536	5 (-6, 13)	0.30 (-0.40, 0.83)
Chengdu	32593	-54 (-189, 59)	-0.17 (-0.58, 0.18)
Dazhou	4132	-5 (-18, 7)	-0.13 (-0.43, 0.17)
Deyang	3819	14 (-17, 40)	0.37 (-0.44, 1.05)
Guangan	4550	9 (-22, 26)	0.19 (-0.49, 0.58)
Guangyuan	3375	-10 (-35, 11)	-0.28 (-1.04, 0.34)
Leshan	5834	23 (-25, 65)	0.39 (-0.43, 1.12)
Luzhou	5418	9 (-4, 18)	0.16 (-0.07, 0.32)
Meishan	4390	26 (-10, 52)	0.60 (-0.22, 1.18)
Mianyang	8958	101 (54, 138)	1.13 (0.60, 1.54)
Nanchong	4130	41 (24, 55)	0.98 (0.57, 1.32)
Neijiang	5601	63 (35, 86)	1.12 (0.63, 1.54)
Suining	5539	37 (21, 50)	0.66 (0.38, 0.90)
Yibin	4477	34 (19, 47)	0.75 (0.43, 1.04)
Ziyang	2397	14 (-6, 28)	0.58 (-0.26, 1.17)
Zigong	4883	29 (16, 40)	0.60 (0.33, 0.82)
Southwestern Sichuan Mountain (Subtropical Monsoon Climate)			
Liangshan	12557	-26 (-88, 32)	-0.20 (-0.70, 0.25)
Panzhihua	3547	7 (-20, 23)	0.19 (-0.57, 0.65)
Yaan	1435	4 (-7, 11)	0.28 (-0.46, 0.78)
Northwestern Sichuan Plateau (Alpine Climate)			
Aba	2512	-7 (-26, 9)	-0.29 (-1.04, 0.36)
Ganzi	2919	-3 (-9, 3)	-0.09 (-0.32, 0.11)

[a] City-specific cumulative effects used here were obtained from the BLUP of meta-regression after controlling for the effect modifiers.

Table S6: Sensitivity analysis for attributable morbidity risk during the study period

Modelling Choices	DF	Attributable diarrheal cases	Attributable fraction
Relative humidity	2	304 (105, 450)	0.24 (0.08, 0.36)
	3	310 (102, 456)	0.25 (0.08, 0.37)
	4	310 (113, 466)	0.25 (0.09, 0.37)
	5	325 (130, 475)	0.26 (0.10, 0.38)
Temperature	2	308 (92, 446)	0.25 (0.07, 0.36)
	3	310 (109, 479)	0.25 (0.09, 0.38)
	4	307(101, 464)	0.25 (0.08, 0.37)
	5	303 (92, 463)	0.24 (0.07, 0.37)
Lag-response dimension	2	373 (172, 511)	0.30 (0.14, 0.41)
	3	317 (117, 477)	0.25 (0.09, 0.38)
	4	310 (129, 470)	0.25 (0.10, 0.38)
	5	240 (9, 392)	0.19 (0.01, 0.31)
